# Reporting categories in urine test strip analysis: Croatian survey and call for action

**DOI:** 10.11613/BM.2019.020709

**Published:** 2019-06-15

**Authors:** Ana Dojder, Dora Vuljanić, Valentina Špoljarić, Andrea Saračević, Lora Dukić, Jasna Leniček-Krleža, Jelena Vlašić-Tanasković, Ivana Maradin, Ana Grzunov, Ana-Maria Šimundić

**Affiliations:** 1 Department of Medical Laboratory Diagnostics, University Hospital “Sveti Duh”, Zagreb, Croatia; 2 Department of Laboratory Diagnostics, Children’s Hospital Zagreb, Zagreb, Croatia; 3 Department of Laboratory Diagnostics, General Hospital Pula, Pula, Croatia; 4 Medical-biochemistry Laboratory “Mirjana Plavetić and Ivana Maradin”, Karlovac, Croatia; 5 Croatian Centre for Quality Assessment in Laboratory Medicine (CROQALM), Croatian Society of Medical Biochemistry and Laboratory Medicine, Zagreb, Croatia; 6 Faculty of Pharmacy and Biochemistry, University of Zagreb, Zagreb, Croatia

**Keywords:** urine, test strips, reporting, harmonization

## Abstract

**Introduction:**

In line with the national recommendations, Croatian medical laboratories report urine test strip qualitative analysis results using a categorized scale with defined number of categories. Since concentration ranges for measured analytes have not been provided by national professional authority, it is up to the laboratories to define their own categories. The aim of study was to assess the comparability of concentrations assigned to different categories used in reporting the results of dipstick urinalysis in Croatian laboratories.

**Material and methods:**

A questionnaire was e-mailed to all Croatian medical laboratories (N = 195). They were asked to provide the number of categories and respective concentrations for each parameter. Data were described as numbers and percentages. Values above the upper reference range limit, which were assigned as normal and/or trace category, were considered as false negative.

**Results:**

Response rate was 71% (139/195). Seventy percent (98/139) of laboratories report their results with either higher (77/98; 79%) or lower (2/98; 2%) number of categories, relative to the national recommendation, whereas 19/98 (19%) report their results as concentrations. Great heterogeneity of reporting categories was observed. Multiple categories were assigned to same concentrations and there was a large overlap of concentrations for most categories. Considerable proportion of laboratories reported false negative results for ketones (42%), leukocytes (30%) and glucose (21%).

**Conclusions:**

The concentrations assigned to categories used to report the results of dipstick urinalysis are not comparable among Croatian medical laboratories. There is an urgent need for harmonization and standardization of reporting the results of urine dipstick analysis in Croatia.

## Introduction

Urine test strip analysis is a semi-quantitative analysis in which the concentration of an analyte is measured in urine and expressed as a category. Depending on the manufacturer, the number of measurement fields (*i.e.* strip pad) on the test strip *per* analyte may vary. Most test strips have one field for negative result and various number of fields for positive results, in an increasing order of the concentration of an analyte. Thus, most analytes measured by urine test strips have more measurement fields then categories commonly used to report test results. It is often up to the medical laboratory to define their own categories and respective concentration ranges.

In Croatia, in line with the recommendation of the Croatian Chamber of Medical Biochemists (CCMB), medical laboratories report the results of urine test strip qualitative analysis using the categorized scale with a defined number of categories (Table 1) (*1*). However, respective concentrations or concentration ranges for measured analytes have not been provided by CCMB and it is thus, up to the laboratory to decide which category to assign to particular concentration of analyte in urine.

**Table 1:** Categories for reporting results of test strips urinalysis recommended by the Croatian Chamber of Medical Biochemists

**Parameter**	**Units**		**Reference range**		
	**Arbitrary units**	**SI units**	**Age/sex** **(male, female)**	**Range**	
				**Arbitrary units**	**SI units**
	/	pH units	newborns	/	5.0 - 7.0
**pH**			children		4.5- 8.0
			adults		5.0 - 9.0
	/	kg/L	newborns	/	1.001 - 1.021
**specific gravity**			children		1.002 - 1.006
			adults		1.002 - 1.030
**glucose**	norm - 3/+++	mmol/L	newborns, children, adults	norm	< 0.8
**bilirubin**	0/neg - 3/+++	µmol/L	newborns, children, adults	0/neg.	0
**ketones**	0/ne - 3/+++	mmol/L	newborns, children, adults	0/neg	< 0.5
**erythrocytes/haemoglobin**	0/neg. - 3/+++	Erc x 10^6^/L	newborns, children, adults	0/neg	< 10
**proteins**	0/neg - 3/+++	g/L	newborns	0/neg	< 0.3
			children, adults	0/neg	< 0.2
**urobilinogen**	norm - 3/+++	µmol/L	newborns, children, adults	norm	< 17
**nitrites**	0/neg - 2/++	µmol/L	newborns, children, adults	0/neg	0
**leukocyte esterase**	0/neg - 3/+++	Lkc x10^6^/L	newborns, children	0/neg	< 25
			adults	0/neg	< 10
Norm – normal. Neg – negative.					

Moreover, most external quality providers (*e.g.* Reference Institute for Bioanalytics (RfB), Institut für Standardisierung und Dokumentation im medizinischen Laboratorium (INSTAND), Labquality) also use categorized scale for reporting urine test strip qualitative analysis results, without declaring accompanying concentrations or concentration ranges. Consistent assignment of urine test strips categories to the respective concentration or concentration ranges of measured analytes is not only a crucial prerequisite for comparability of results between medical laboratories, but also for a longitudinal comparability of results in the same laboratory. Latter is especially significant if different test strips are used interchangeably over time. Given the fact that urine analysis is often a first step in the diagnostic pathway of a wide range of diseases, such inconsistency and lack of standardization may lead to patient misclassification and thus affect patient safety (*2*-*8*).

To the best of our knowledge, nobody has so far investigated the level of agreement between concentrations (or concentration ranges) assigned to different reporting categories of the urine test strips, among different medical laboratories. The size of this potential problem is thus unknown and yet remains to be explored.

The aim of our study was therefore to assess the comparability of concentrations assigned to different categories that are used to report the urine test strip analysis results in Croatian medical laboratories.

## Materials and methods

### Questionnaire

**Figure 1:**
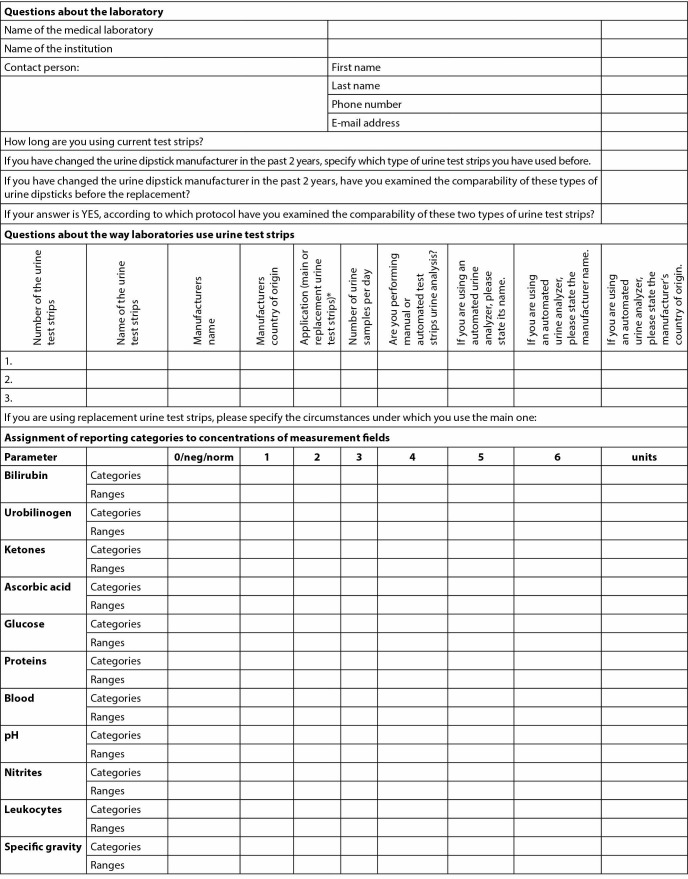
Questionnaire sent to all Croatian medical laboratories.

A questionnaire was e-mailed to all Croatian medical laboratories (N = 195) in March 2017 and it contained questions about the laboratory (size, type, number of urine analysis *per* day, *etc*.) and questions about the way laboratories use urine test strips (Figure 1). Medical laboratories were also asked to provide the number of categories and respective concentrations or concentration ranges for each test strip parameter (Figure 1). If medical laboratories were using more than one test strip (two brands from two different manufacturers or two types of strips of the same manufacturer), they were asked to fill in the requested information for each strip they use, separately. Detailed instructions on how to complete the survey were provided with the questionnaire.

### Statistical analysis

Data were reported as numbers and percentages. Medical laboratories that report urine test strip results as concentrations or concentration ranges were not included in data analysis. We only presented data for those medical laboratories that report the results on a categorized scale. We considered +/- as trace. To evaluate classification accuracy, categories were evaluated relative to the reference ranges for each parameter. In cases when normal and/or trace category was assigned to concentrations above the upper reference range limit, such result was considered as false negative.

Parameters included in this analysis were: glucose, proteins, leukocytes, bilirubin, urobilinogen, erythrocytes and ketones. Since specific gravity and pH are reported as quantitative values (not categories), they were excluded from our study. Also, nitrite results weren’t included because test strips for nitrites commonly have only one negative and 1-2 positive fields, depending on different manufacturer. Data for ascorbic acid were also not statistically analysed because this parameter has no clinical relevance and is just used to confirm or exclude the interference caused by it. Microsoft Excel (MS Office) was used for statistical analysis.

## Results

### Participating medical laboratories and how they use test strips

Response rate in our study was 71% (139/195). Out of 139 medical laboratories which have provided their replies to survey (Figure 1), 121 (87%) answered basic questions about urine test strips they use (Figure 1). It should be noted that not all of 139 medical laboratories provided their replies about categories assignment for all parameters. Therefore, the number of responding medical laboratories varies among each urine parameter. The most commonly used type of urine dipsticks are listed in Table 2. In 90% of the participating medical laboratories, the average number of urine samples *per* day is ≤ 150. Out of 121 medical laboratories who have replied to basic questions about urine dipsticks, 78 of them (64%) use automated urine analyser, while others read urine test strips manually (visually reading). Majority of medical laboratories (52%) use the same test strip (same manufacturer) for more than 5 years, whereas almost 25% of them use one brand of test strips for not more than 2 years. When asked about the number of test strips type in use at the same time in their medical laboratory, 17% (20/121) of laboratories declared to use more than one type of urine test strips, but only 13/20 provided reasons for this. Of those who declared the reasons for using more than one test strip at the same time in their laboratory, 4/13 use both test strip brands interchangeably without preference for one over another, 4/13 use a second brand of test strips just in case of a failure of the automated urine analyser and 8/13 use it for sample retesting and/or confirmation of the result. Three laboratories provided more than one reason for using different types of urine test strips. Only 14/121 (12%) laboratories answered the question whether they changed the type of urine dipsticks in the last 2 years. Ten of them claimed to have tested the comparability of the old and new test strips before replacement wherein only three of them have specified which procedure they have used.

**Table 2:** The list of test strips and their manufacturers used in Croatian medical laboratories

**Type of test strips used in Croatian medical laboratories in 2017 (N = 121)**	**Frequency N (%)**
Multistix 10 SG (Siemens, München, Germany)	32 (26)
Combur 10 Test M (Roche, Basel, Switzerland)	30 (25)
iChem Velocity (Beckman Coulter, Brea, California, USA)	20 (17)
Combi Screen 11 SYS (Analyticon, Lichtenfels, Germany)	15 (12)
Urignost 11 (Biognost, Zagreb, Croatia)	11 (9.1)
Combi Screen 10SL (Analyticon, Lichtenfels, Germany)	7 (5.8)
Combo Stik 10M (DFI co. Ltd, Gyeongsangnam-do, South Korea)	6 (5.0)
Choice Line 10 (Roche, Basel, Switzerland)	5 (4.1)
Combina 10M (Human, Wiesbaden, Germany)	5 (4.1)
Chronocomb 11 SYS (Analyticon, Lichtenfels, Germany)	2 (1.7)
LabStrip U11 Plus (77 Elektronika, Budapest, Hungary)	2 (1.7)
Clinitek Novus (Siemens, München, Germany)	1 (0.83)
Combina 13 (Human, Wiesbaden, Germany)	1 (0.83)
Combur 10 Test UX (Roche, Basel, Switzerland)	1 (0.83)
Keto-Diastix (Bayer, Leverkusen, Germany)	1 (0.83)
Meditape (Sysmex, Kobe, Japan)	1 (0.83)
Medi-Test Uryxxon 10, (Macherey-Nagel, Düren, Germany)	1 (0.83)
URIN-10 (Spinreact, Girona, Spain)	1 (0.83)
Cobas U pack (Roche, Basel, Switzerland)	1 (0.83)

### Assignment of categories to different concentrations or concentration ranges of urine test strip parameters

Out of 139 responding medical laboratories, 98 (70%) of them do not report urine test strip results in accordance with the CCMB recommendation. Out of those medical laboratories which do not report urine test strip results in accordance with the CCMB recommendation, 77/98 (79%) report more categories than recommended, 2/98 (2%) report less categories and 19/98 (19%) report results as concentrations or concentration ranges. Latter were excluded from further analysis.

#### Glucose

Among 119 responding medical laboratories, up to 7 categories (normal, trace, 1+, 2+, 3+, 4+ and 5+) were used to report urinary test strip glucose results in the concentration range of 1.4 - 111 mmol/L (Supplementary Table 1). The largest differences of assigned reporting categories were at 5.6 and 56 mmol/L. Responding laboratories have assigned up to 4 categories for test strip fields at 5.6 mmol/L (normal, trace, 1+ and 2+), as well as at 56 mmol/L (2+, 3+, 4+ and 5+) (Table 3).

**Table 3:** Glucose concentrations and corresponding categories assigned by 119 medical laboratories

**Glucose concentration (mmol/L)**															
**N**	**1.4**	**2.8**	**3.9**	**5.6**	**8.3**	**11**	**14**	**15**	**17**	**28**	**30**	**56**	**60**	**110**	**111**
N = 119/119	Trace= 2/2	N = 2/76	2+ = 1/1	N = 6/115	2+ = 3/3	1+ = 2/19	1+ = 24/47	1+ = 12/13	2+ = 8/37	2+ = 31/69	2+ = 13/13	2+ = 4/105	2+ = 1/13	3+ = 6/13	3+ = 16/24
		trace = 10/76		trace = 19/115		2+ = 16/19	2+ = 11/47	2+ = 1/13	3+ = 29/37	3+ = 32/69		3+ = 63/105	3+ = 12/13	4+ = 7/13	4+ = 8/24
		1+ = 64/76		1+ = 47/115		3+ = 1/19	3+ = 12/47			4+ = 6/69		4+ = 36/105			
				2+ = 43/115								5+ = 2/105			
N – normal. Note: The number of medical laboratories that assigned certain categories to different glucose concentrations are represented in the rows.															

Table 3 lists all the concentrations found on the urine test strips used in Croatian medical laboratories. The rows show how many laboratories have assigned a certain concentration to each category. For example, 37 laboratories have measurement field for glucose concentration 17 mmol/L. There are 8 laboratories which report this concentration as 2+, while 29 of them report it as 3+ category.

Wide concentration ranges were assigned by participating medical laboratories for each reporting category (Table 4). The greatest declared analytical sensitivity for glucose was 1.4 mmol/L and it was observed in only one type of urine test strip (LabStrip U11 Plus, 77 Elektronika). Concentrations higher than 56 mmol/L can be measured with only some urine dipsticks (Multistix 10 SG, Siemens; Urignost 11, Biognost; Keto-Diastix, Bayer and URIN-10, Spinreact). All participating medical laboratories defined first measurement field as normal glucose result. False negative prevalence was 21%.

**Table 4:** Overlapping of concentration ranges of glucose for all categories assigned by Croatian medical laboratories

**Glucose**	**Glucose concentration (mmol/L)**														
**category**	**1.4**	**2.8**	**3.9**	**5.6**	**8.3**	**11**	**14**	**15**	**17**	**28**	**30**	**56**	**60**	**110**	**111**
**N/trace**															
**1+**															
**2+**															
**3+**															
**4+**															
**5+**															
N - normal. Note: Shades of grey colour represents degrees of reporting categories from normal to 5+ category.															

#### Proteins

Hundred and eighteen medical laboratories used 15 different types of urine test strips to report 0.10-20 g/L concentrations of urinary proteins as 6 categories (negative, trace, 1+, 2+, 3+ and 4+) (Supplementary Table 2). The choice of concentrations on test strips differed between manufacturers. While some protein concentrations were quite common on test strips from various manufacturers (*e.g.* most test strips used in our study had 0.3 and 1 g/L as measurement fields), some were less frequent or even present in only one manufacturer. For example, 0.10, 0.50 and 6 g/L were available as a field only on iChem Velocity test strips (Beckman Coulter), whereas 0.25, 0.75 and 1.5 g/L were available as a field only on Combur 10 Test M (Roche). The greatest analytical sensitivity declared by manufacturer is 0.10 g/L and it was achieved with only one type of urine dipsticks (iChem Velocity, Beckman Coulter). All medical laboratories assigned first measurement field to negative category of urinary proteins. Large differences in assigned categories were observed for most test strip fields (Table 5) and wide concentration ranges were observed for each reporting category (Table 6). Only one of 118 medical laboratories reported false negative results.

**Table 5:** Urine proteins concentrations and corresponding categories assigned by 118 medical laboratories

**Protein concentration (g/L)**													
**N**	**0.10**	**0.15**	**0.25**	**0.30**	**0.50**	**0.75**	**1**	**1.50**	**3**	**5**	**6**	**10**	**20**
N = 118/118	N = 6/19	N = 8/63	1+ = 6/6	trace = 1/94	1+ = 19/19	2+ = 6/6	1+ = 6/113	2+ = 1/6	2+ = 14/68	3+ = 46/50	3+ = 11/19	3+ = 6/12	3+ = 21/37
	trace = 10/19	trace = 30/63		1+ = 92/94			2+ = 106/113	3+ = 5/6	3+ = 54/68	4+ = 4/50	4+ = 8/19	4+ = 6/12	4+ = 16/37
	1+ = 3/19	1+ = 25/63		2+ = 1/94			3+ = 1/113						
N - negative. Note: The number of medical laboratories that assigned certain categories to different protein concentrations are represented in the rows.													

**Table 6:** Overlapping of concentration ranges of proteins for all categories assigned by Croatian medical laboratories

**Protein category**	**Protein concentration (g/L)**												
	**0.10**	**0.15**	**0.25**	**0.30**	**0.50**	**0.75**	**1**	**1.5**	**3**	**5**	**6**	**10**	**20**
**N/trace**													
**1+**													
**2+**													
**3+**													
**4+**													
N - negative. Note: Shades of grey colour represents degrees of reporting categories from negative to 4+ category.													

#### Leukocytes

Leukocyte counts of 10-500 Lkc x10^6^/L were reported as 6 categories (negative, trace, 1+, 2+, 3+ and 4+) depending on each participating medical laboratory (N = 121) (Supplementary Table 3). Participating medical laboratories used 16 different types of urine test strips and defined their first measurement field as negative. The greatest analytical sensitivity declared by manufacturer (10 Lkc x10^6^/L) was achieved with three types of urine dipsticks (Combina 10 M, Human; Combur 10 Test M and Combur 10 Test UX, Roche). While some leukocyte counts were detectable by test strips from various manufacturer (*e.g.* all urine test strips had measurement field for 500 Lkc x10^6^/L), some were less frequent or present in only one type of urine test strips (250 Lkc x10^6^/L, iChem Velocity, Beckman Coulter). The most pronounced difference in assigned categories was noticed at 15 and 25 Lkc x10^6^/L leukocyte count which were reported as three different categories (Table 7). The false negative prevalence was 30%. Reporting categories showed large overlap (Table 8).

**Table 7:** Leukocyte counts and corresponding categories assigned by 121 medical laboratories

**Leukocyte count (Lkc x10^6^/L)**								
**N**	**10-25**	**15**	**25**	**70**	**75**	**125**	**250**	**500**
N=121/121	1+ = 27/27	N = 1/43	N = 1/50	1+ = 35/39	1+ = 15/82	2+ = 37/44	2+ = 15/20	3+ = 119/121
		trace = 18/43	trace = 7/50	2+ = 4/39	2+ = 67/82	3+ = 7/44	3+ = 5/20	4+ = 2/121
		1+ = 24/43	1+ = 42/50					
N - negative. Note: The number of medical laboratories that assigned certain categories to different leukocyte counts are represented in the rows.								

**Table 8:** Overlapping of leukocyte count ranges for all categories assigned by Croatian medical laboratories

**Leukocytes category**	**Leukocyte count (Lkc x10^6^/L)**							
	**10-25**	**15**	**25**	**70**	**75**	**125**	**250**	**500**
**N/trace**								
**1+**								
**2+**								
**3+**								
**4+**								
N - negative. Note: Shades of grey colour represents degrees of reporting categories from negative to 4+ category.w								

#### Bilirubin

Eighty three medical laboratories used even up to 8 categories (negative, trace, 1+, 2+, 3+, 4+, 5+ and 6+) to report urinary bilirubin concentrations in range of 8.5-103 µmol/L (Supplementary Table 4). Only one out of 10 types of analysed urine test strips could be used to measure the lowest bilirubin concentration of 8.5 µmol/L (iChem Velocity, Beckman Coulter). All medical laboratories defined their first measurement field as negative bilirubin result. The most heterogeneity in reporting categories was observed for the test strip field assigned to 100 µmol/L (Table 9). Thirteen out of 83 medical laboratories reported false negative results. Considerable overlap in categories is shown, wherein the widest concentration range of 8.5 - 100 µmol/L was perceived at 1+ category (Table 10).

**Table 9:** Bilirubin concentrations and corresponding categories assigned by 83 medical laboratories

**Bilirubin concentration (µmol/L)**									
**N**	**8.5**	**17**	**34**	**35**	**50**	**51**	**70**	**100**	**103**
N = 83/83	N = 7/19	N = 3/83	1+ = 9/19	2+ = 35/35	1+ = 2/45	2+ = 3/3	1+ = 1/55	1+ = 1/44	3+ = 3/3
	trace = 3/19	1+ = 78/83	2+ = 8/19		2+ = 41/45		2+ = 12/55	2+ = 1/44	
	1+ = 9/19	2+ = 2/83	3+ = 2/19		3+ = 1/45		3+ = 41/55	3+ = 40/44	
					4+ = 1/45		5+ = 1/55	4+ = 1/44	
								6+ = 1/44	
N- negative. Note: The number of medical laboratories that assigned certain categories to different bilirubin concentrations are represented in the rows.									

**Table 10:** Overlapping of concentration ranges of bilirubin for all categories assigned by Croatian medical laboratories

**Bilirubin category**	**Bilirubin concentration (µmol/L)**								
	**8.5**	**17**	**34**	**35**	**50**	**51**	**70**	**100**	**103**
**N**									
**trace**									
**1+**									
**2+**									
**3+**									
**4+**									
**5+**									
**6+**									
N - negative. Note: Shades of grey colour represents degrees of reporting categories from negative to 6+ category.									

#### Urobilinogen

Sixteen different urine test strips were used by 110 medical laboratories to report 16-200 µmol/L concentrations of urobilinogen as normal, trace, 1+, 2+, 3+, 4+ and 5+ categories (Supplementary Table 5). The lowest urobilinogen concentration that can be measured is 16 µmol/L which can be detected by two types of urine dipsticks made by the same manufacturer (Multistix 10 SG and Clinitek Novus, Siemens). The number of categories assigned to concentrations that can be determined with 16 types of urine dipsticks included in our study, varied from 2 to 4. The greatest number of categories was assigned to the measurement field at 140 µmol/L (Table 11). The false negative prevalence was 9.1%. There were wide concentration ranges for each reporting category, *e.g.* 16-70 for 1+ and 33-140 µmol/L for 2+ category (Table 12). All 110 participating medical laboratories assigned their first measurement field to normal result of urobilinogen.

**Table 11:** Urobilinogen concentrations and corresponding categories assigned by 110 medical laboratories

**Urobilinogen concentration (µmol/L)**														
**N**	**16**	**17**	**33**	**34**	**35**	**50**	**51**	**66**	**70**	**100**	**103**	**131**	**140**	**200**
N = 110/110	N = 14/21	N = 1/45	1+ = 4/6	N = 5/21	trace = 2/31	1+ = 17/20	1+ = 2/46	2+ = 2/4	1+ = 5/60	2+ = 15/20	2+ = 4/46	3+ = 15/29	2+ = 6/59	3+ = 25/41
	trace= 1/21	trace = 2/45	2+ = 2/6	1+ = 15/21	1+ = 22/31	2+ = 3/20	2+ = 39/46	3+ = 2/4	2+ = 47/60	3+ = 4/20	3+ = 41/46	4+ = 14/29	3+ = 49/59	4+ = 15/41
	1+ = 6/21	1+ = 42/45		2+ = 1/21	2+ = 7/31		3+ = 5/46		3+ = 8/60	4+ = 1/20	4+ = 1/46		4+ = 3/59	5+ = 1/41
													5+ = 1/59	
N - normal. Note: The number of medical laboratories that assigned certain categories to different urobilinogen concentrations are represented in the rows.														

**Table 12:** Overlapping of concentration ranges of urobilinogen for all categories assigned by Croatian medical laboratories

**Urobilinogen**	**Urobilinogen concentration (µmol/L)**													
**category**	**16**	**17**	**33**	**34**	**35**	**50**	**51**	**66**	**70**	**100**	**103**	**131**	**140**	**200**
**N**														
**trace**														
**1+**														
**2+**														
**3+**														
**4+**														
**5+**														
N - normal. Note: Shades of grey colour represents degrees of reporting categories from normal to 5+ category.														

#### Erythrocytes

Among 84 participating medical laboratories, erythrocyte counts of 5-326 Erc x10^6^/L were reported as 7 categories (negative, trace, 1+, 2+, 3+, 4+ and 5+) (Supplementary Table 6). Nine out of 15 included types of urine test strips had the same declared analytical sensitivity of 5 Erc x10^6^/L. All included medical laboratories assigned their first measurement field to negative result of erythrocytes. The greatest difference of assigned categories was noticed at 10 and 250 Erc x10^6^/L concentrations which were defined by three different categories (Table 13). Twenty nine out of 84 medical laboratories reported false negative results. iChem Velocity (Beckman Coulter) urine dipsticks differed from others because they report their results as concentrations of hemoglobin rather than erythrocyte count and therefore these hemoglobin concentrations were converted to approximate erythrocyte counts calculated by mean of reference range of MCH (30.65 pg). Therefore, 33, 163 and 326 Erc x10^6^/L were measured only with this type of urine dipsticks. Ranges of erythrocyte counts for each reporting category are largely overlapping (Table 14).

**Table 13:** Erythrocyte count and corresponding categories assigned by 84 medical laboratories

**Erythrocyte count (Erc x10^6^/L)**												
**N**	**5-10**	**10**	**25**	**33**	**50**	**80**	**150**	**163**	**200**	**250**	**300**	**326**
N = 103/103	1+ = 31/31	N = 5/77	1+ = 27/52	1+ = 16/19	2+ = 39/61	2+ = 22/23	3+ = 2/6	2+ = 16/19	3+ = 23/23	3+ = 28/39	3+ = 21/21	3+ = 18/19
		trace = 24/77	2+ = 25/52	2+ = 3/19	3+ = 22/61	3+ = 1/23	4+ = 4/6	3+ = 3/19		4+ = 7/39		4+ = 1/19
		1+ = 48/77								5+ = 4/39		
N - negative. Note: The number of medical laboratories that assigned certain categories to different erythrocyte counts are represented in the rows.												

**Table 14:** Overlapping of erythrocyte count ranges for all categories assigned by Croatian medical laboratories

**Erythrocytes**	**Erythrocyte count (Erc x10^6^/L)**											
**category**	**5-10**	**10**	**25**	**33**	**50**	**80**	**150**	**163**	**200**	**250**	**300**	**326**
**N/trace**												
**1+**												
**2+**												
**3+**												
**4+**												
**5+**												
N - negative. Note: Shades of grey colour represents degrees of reporting categories from negative to 5+ category.												

#### Ketones

One hundred and nineteen medical laboratories used 6 categories (negative, trace, 1+, 2+, 3+ and 4+) to report 0.5-30 mmol/L ketones concentrations (Supplementary Table 7). All of them defined their first measurement field as negative result. Concentration level of 0.5 mmol/L represents the greatest analytical sensitivity declared by manufacturer. Nine out of 16 analysed urine test strips could detect this concentration of ketones in urine. All concentrations that can be measured with included types of urine test strips were reported as at least two and quite often with three different categories (Table 15). The false negative prevalence was 42%. Concentration ranges of reporting categories are overlapping (Table 16).

**Table 15:** Ketones concentrations and corresponding categories assigned by 119 medical laboratories

**Ketones concentration (mmol/L)**													
**N**	**0.5**	**1**	**1.5**	**2**	**2.5**	**4**	**5**	**6**	**8**	**10**	**15**	**16**	**30**
N = 119/119	N = 10/71	N = 1/59	1+ = 47/53	1+ = 16/19	1+ = 21/22	1+ = 2/64	2+ = 25/33	1+ = 1/19	2+ = 8/59	2+ = 21/46	3+ = 41/53	3+ = 36/57	3+ = 22/22
	trace = 33/71	trace = 11/59	2+ = 6/53	2+ = 3/19	2+ = 1/22	2+ = 59/64	3+ = 8/33	2+ = 16/19	3+ = 48/59	3+ = 23/46	4+ = 12/53	4+ = 21/57	
	1+ = 28/71	1+ = 47/59				3+ = 3/64		4+ = 2/19	4+ = 3/59	4+ = 2/46			
N - negative. Note: The number of medical laboratories that assigned certain categories to different ketones concentrations are represented in the rows.													

**Table 16:** Overlapping of concentration ranges of ketones for all categories assigned by Croatian medical laboratories

**Ketones**	**Ketones concentration (mmol/L)**												
**category**	**0.5**	**1**	**1.5**	**2**	**2.5**	**4**	**5**	**6**	**8**	**10**	**15**	**16**	**30**
**N / trace**													
**1+**													
**2+**													
**3+**													
**4+**													
N- negative. Note: Shades of grey colour represents degrees of reporting categories from negative to 4+ category.													

## Discussion

Our results point to the lack of harmonization and comparability of reporting qualitative urine test strip results among Croatian laboratories. Furthermore, our study shows that concentrations assigned to categories used to report results of the urine dipsticks analysis differ substantially among Croatian medical laboratories. Multiple categories are assigned to same concentrations or concentration ranges and there is a large range of concentrations for most categories, for all test strip parameters included in this study. In general, higher number of measurement fields results in greater heterogeneity of reporting categories. We have also observed that the majority of the participating medical laboratories are not using categories recommended by CCMB.

This is a serious problem which compromises patient safety and improvement is necessary. Substantial proportion of medical laboratories (17%) in our study have declared to use more than one type of urine dipsticks interchangeably, which could affect the longitudinal patient monitoring in the same medical laboratory, in cases where test strips are not comparable.

Although the use of urine dipsticks in diagnosis and treatment decisions of numerous disorders is being more and more discouraged recently, some guidelines still recommend urine dipstick analysis as an important step in the diagnostic algorithm. For example, American Urology Association (AUA) recommends that blood positive urine dipstick with negative sediment count should be followed by three additional sediment estimations and if one of them is positive further processing, e.g. some more expensive and invasive diagnostic methods (cystoscopy and/or imaging) is required (*2*). Also, National Institution for Health and Care Excellence (NICE) and Kidney Health Australia guidelines for assessment and management of chronic kidney disease (CKD) recommend urine dipsticks rather than microscopy for haematuria confirmation (*3*, *4*).

Furthermore, several guidelines for management and treatment of urinary tract infections (UTI) recommend that negative urine dipsticks are used to exclude UTI, whereas positive leukocytes, erythrocytes and nitrites indicate possible UTI (*5*-*8*). Leukocytes measured by urine dipsticks are also used as a biomarker of periprosthetic joint infection, where 2+ for leukocyte esterase is considered as one of five minor criteria for the diagnosis of periprosthetic joint infection (*9*, *10*).

Obviously, variations in the number of cells or concentration of a certain parameter assigned to various categories, may affect the accuracy of diagnosis in a variety of clinical conditions, as indicated above. Such heterogeneity hence, may cause diagnostic errors (misdiagnosis, missed diagnosis), lead to wrong medical decisions and affect patient safety and outcome.

We wish to point out that a considerable proportion of medical laboratories in Croatia report false negative results for ketones (42%), leukocytes (30%) and glucose (21%). These medical laboratories report results above the cut-off values defined by CCMB, as negative, thus jeopardizing that patients with various disorders remain undetected by urine dipstick urinalysis due to incorrectly assigned concentrations to reporting categories.

It is our belief that the level of awareness for this issue is generally very low among laboratory professionals and healthcare workers in general. How well are indeed Croatian medical laboratories and their users aware of the heterogeneity observed in our study, remains to be investigated in some future study. Nevertheless, we believe that immediate action is warranted to educate the users of medical laboratory services of the limitations of urine dipstick analysis and lack of standardization in reporting dipstick analysis results. Moreover, it is up to professional bodies in Croatia to deal with this problem by providing clear and unambiguous guidance on reporting urinary dipstick analysis results.

Our study was a self-reported survey. It is therefore possible that survey responses deviate from the truth and that our results are not accurately describing the real situation. This is, for sure, one possible limitation of our study. Nevertheless, we do believe that our study may at least to some extent indicate the size of the problem and raise awareness of laboratory professionals for this issue.

In conclusion, our study shows that the concentrations assigned to categories used to report the results of the dipstick urinalysis in Croatian medical laboratories are not comparable among laboratories. Furthermore, CCMB recommendations for reporting urine dipstick analysis are not widely accepted by Croatian medical laboratories. There is an urgent need for harmonization and standardization of reporting the results of urine dipstick analysis in Croatia. We hope that our study would raise the awareness of this unrecognised issue.

## Supplementary material

Supplementary tables
