# Supplementary material for: Reporting categories in urine test strip analysis: Croatian survey and call for action
Source: Biochem Med (Zagreb). 2019 Jun 15;29(2):020709. doi: 10.11613/BM.2019.020709 (PMC6559610; doi:10.11613/BM.2019.020709)
Supplement: Supplementary file 1 — Supplementary tables [file bm-29-2-020709-S1.pdf]

Supplementary materials

Table 1. Glucose concentrations and assigned categories depending on the urine dipstick type reported by 119 medical laboratories

Urine dipstick type	Manufacturer	Glucose concentration (mmol/L)															
		N	1.4	2.8	3.9	5.6	8.3	11	14	15	17	28	30	56	60	110	111
Multistix 10 SG	Siemens	N=23/23	/	/	/	N = 5/23<br>trace = 10/23<br>1+ = 8/23	/	/	1+ = 21/23<br>2+ = 2/23	/	/	2+ = 22/23<br>3+ = 1/23	/	2+ = 4/23<br>3+ = 19/23	/	/	3+ = 15/23<br>4+ = 8/23
Combur 10 Test M	Roche	N=21/21	/	1+ = 21/21	/	1+ = 4/21<br>2+ = 17/21	/	/	/	/	2+ = 4/21<br>3+ = 17/21	/	/	3+ = 11/21<br>4+ = 10/21	/	/	/
iChem Velocity	Beckman Coulter	N=19/19	/	N = 2/19<br>trace = 6/19<br>1+ = 11/19	/	trace = 1/19<br>1+ = 15/19<br>2+ = 3/19	/	1+ = 2/19<br>2+ = 16/19<br>3+ = 1/19	/	/	/	2+ = 4/19<br>3+ = 15/19	/	3+ = 10/19<br>4+ = 9/19	/	/	/
Combi Screen 11 SYS plus	Analyticon	N=13/13	/	trace = 3/13<br>1+ = 10/13	/	trace = 1/13<br>1+ = 6/13<br>2+ = 6/13	/	/	1+ = 1/13<br>2+ = 6/13<br>3+ = 6/13	/	/	2+ = 3/13<br>3+ = 8/13<br>4+ = 2/13	/	3+ = 9/13<br>4+ = 3/13<br>5+ = 1/13	/	/	/
Urignost 11	Biognost	N=12/12	/	/	/	N = 1/12<br>trace = 5/12<br>1+ = 6/12	/	/	/	1+ = 11/12<br>2+ = 1/12	/	/	2+ = 12/12	/	2+ = 1/12<br>3+ = 11/12	3+ = 6/12<br>4+ = 6/12	/
Combi Screen 10SL	Analyticon	N=7/7	/	1+ = 7/7	/	1+ = 1/7<br>2+ = 6/7	/	/	2+ = 1/7<br>3+ = 6/7	/	/	3+ = 3/7<br>4+ = 4/7	/	3+ = 2/7<br>4+ = 4/7<br>5+ = 1/7	/	/	/
Combo Stik 10M	DFI co. Ltd	N=7/7	/	1+ = 7/7	/	2+ = 7/7	/	/	/	/	3+ =7/7	/	/	3+ =2/7<br>4+ = 5/7	/	/	/
Choice Line 10	Roche	N=5/5	/	/	/	1+ = 4/5<br>2+ = 1/5	/	/	/	/	2+ = 4/5<br>3+ = 1/5	/	/	3+ =4/5<br>4+ = 1/5	/	/	/
Clinitek Novus	Siemens	N=1/1	/	/	/	trace = 1/1	/	/	1+ = 1/1	/	/	2+ = 1/1	/	3+ = 1/1	/	/	/
LabStrip U11 Plus	77 Elektronika	N=2/2	trace = 2/2	1+ = 2/2	/	/	2+ = 2/2	/	/	/	/	3+ = 2/2	/	3+ =1/2<br>4+ = 1/2	/	/	/
Chronocomb 11 SYS	Analyticon	N=1/1	/	trace = 1/1	/	1+ = 1/1	/	/	2+ = 1/1	/	/	3+ = 1/1	/	4+ = 1/1	/	/	/
Medi-Test Uryxxon 10	Macherey-Nagel	N=1/1	/	1+ = 1/1	/	/	2+ = 1/1	/	/	/	/	3+ = 1/1	/	/	/	/	/
Combur 10 Test UX	Roche	N=1/1	/	1+ = 1/1	2+ = 1/1	/	/	/	/	/	3+ = 1/1	/	/	4+ = 1/1	/	/	/
Combina 10M	Human	N=3/3	/	1+ = 3/3	/	2+ = 3/3	/	/	/	/	3+ = 3/3	/	/	3+ = 2/3<br>4+ = 1/3	/	/	/
Combina 13	Human	N=1/1	/	1+ = 1/1	/	1+ = 1/1			2+ = 1/1	/	/	3+ = 1/1	/	3+ = 1/1	/	/	/
Keto-Diastix	Bayer	N=1/1	/	/	/	trace = 1/1	/	/	1+ = 1/1	/	/	2+ = 1/1	/	3+ = 1/1	/	/	3+ = 1/1
URIN-10	Spinreact	N=1/1	/	/	/	1+ = 1/1	/	/	/	1+ = 1/1	/	/	2+ = 1/1	/	3+ = 1/1	4+ = 1/1	/

N - normal. / - this concentration is not measured with this type of urine dipsticks. Shades of grey colour represents degrees of reporting categories from normal to 5+ category.

Table 2. Urine proteins concentrations and assigned categories depending on the urine dipstick type reported by 118 medical laboratories

Urine dipstick type	Manufacturer	Protein concentration (g/L)													
		**N**	**0.10**	**0.15**	**0.25**	**0.30**	**0.50**	**0.75**	**1**	**1.50**	**3**	**5**	**6**	**10**	**20**
Multistix 10 SG	Siemens	N=23/23	/	N = 6/23<br>trace = 12/23<br>1+ = 5/23	/	1+ = 23/23	/	/	1+ = 2/23<br>2+ = 21/23	/	2+ = 7/23<br>3+ = 16/23	/	/	/	3+ = 14/23<br>4+ = 9/23
Combur 10 Test M (manual)	Roche	N=18/18	/	/	/	1+ =18/18	/	/	2+ = 18/18	/	/	3+ =18/18	/	/	/
Combur 10 Test M (analyzer)	Roche	N=6/6	/	/	1+ = 6/6	/	/	2+ = 6/6	/	2+ = 1/6<br>3+ = 5/6	/	3+ = 2/6<br>4+ = 4/6	/	/	/
iChem Velocity	Beckman Coulter	N=19/19	N = 6/19<br>trace = 10/19<br>1+ = 3/19	/	/	/	1+ = 19/19	/	1+ = 2/19<br>2+ = 17/19	/	2+ = 2/19<br>3+ = 17/19	/	3+ = 11/19<br>4+ = 8/19	/	/
Combi Screen 11 SYS plus	Analyticon	N=12/12	/	N = 1/12<br>trace = 5/12<br>1+ = 6/12	/	1+ = 12/12	/	/	2+ = 12/12	/	/	3+ = 12/12	/	/	/
Urignost 11	Biognost	N=12/12	/	trace = 5/12<br>1+ = 7/12	/	trace = 1/12<br>1+ = 11/12	/	/	1+ = 1/12<br>2+ = 11/12	/	2+ = 3/12<br>3+ = 9/12	/	/	/	3+ = 6/12<br>4+ = 6/12
Combi Screen 10SL	Analyticon	N=7/7	/	trace = 3/7<br>1+ = 4/7	/	1+ = 7/7	/	/	2+ = 7/7	/	/	3+ = 7/7	/	/	/
Combo Stik 10M	DFI co.Ltd	N=6/6	/	/	/	1+ = 6/6	/	/	2+ = 6/6	/	3+ = 6/6	/	/	3+ = 2/6<br>4+ = 4/6	/
Choice Line 10	Roche	N=5/5	/	N = 1/5<br>trace = 1/5<br>1+ = 3/5	/	1+ = 4/5<br>2+ = 1/5	/	/	2+ = 4/5<br>3+ = 1/5	/	2+ = 1/5<br>3+ = 4/5	/	/	3+ = 4/5<br>4+ = 1/5	/
Clinitek Novus	Siemens	N=1/1	/	trace = 1/1	/	1+ = 1/1	/	/	2+ = 1/1	/	3+ = 1/1	/	/	4+ = 1/1	/
LabStrip U11 Plus	77 Elektronika	N=2/2	/	trace = 2/2	/	1+ = 2/2	/	/	2+ = 2/2	/	/	3+ = 2/2	/	/	/
Medi-Test Uryxxon 10	Macherey-Nagel	N=1/1	/	/	/	1+ = 1/1	/	/	2+ = 1/1	/	/	3+ = 1/1	/	/	/
Combur 10 Test UX	Roche	N=1/1	/	/	/	1+ = 1/1	/	/	2+ = 1/1	/	/	3+ = 1/1	/	/	/
Combina 10M	Human	N=3/3	/	/	/	1+ = 3/3	/	/	2+ = 3/3	/	/	3+ = 3/3	/	/	/
Combina 13	Human	N=1/1	/	/	/	1+ = 1/1	/	/	1+ = 1/1	/	2+ = 1/1	/	/	/	3+ = 1/1
URIN-10	Spinreact	N=1/1	/	trace = 1/1	/	1+ = 1/1	/	/	2+ = 1/1	/	3+ = 1/1	/	/	/	4+ = 1/1

N - negative. / - this concentration is not measured with this type of urine dipsticks. Shades of grey colour represents degrees of reporting categories from negative to 4+ category.

Table 3. Leukocyte count and assigned categories depending on the urine dipstick type reported by 121 medical laboratories

Urine dipstick type	Manufacturer	Leukocyte count (Lkc x 10^6/L)								
		**N**	**10-25**	**15**	**25**	**70**	**75**	**125**	**250**	**500**
Multistix 10 SG	Siemens	N=25/25	/	N = 1/25<br>trace = 14/25<br>1+ = 10 /25	/	1+ = 24/25<br>2+ = 1/25	/	2+ = 24/25<br>3+ = 1/25	/	3+ = 24/25<br>4+ = 1/25
Combur 10 Test M	Roche	N=24/24	1+ = 24/24	/	/	/	2+ = 24/24	/	/	3+ = 24/24
iChem Velocity	Beckman Coulter	N=20/20	/	/	N = 1/20<br>trace = 7/20<br>1+ = 12/20	/	1+ = 14/20<br>2+ = 6/20	/	2+ = 15/20<br>3+ = 5/20	3+ = 19/20<br>4+ = 1/20
Combi Screen 11 SYS plus	Analyticon	N=13/13	/	/	1+ = 13/13	/	2+ = 13/13	/	/	3+ = 13/13
Urignost 11	Biognost	N=11/11	/	trace = 3/11<br>1+ = 8/11	/	1+ = 9/11<br>2+ = 2/11	/	2+ = 9/11<br>3+ = 2/11	/	3+ = 11/11
Combi Screen 10SL	Analyticon	N=7/7	/	/	1+ = 7/7	/	2+ = 7/7	/	/	3+ = 7/7
Combo Stik 10M	DFI co. Ltd	N=6/6	/	/	1+ = 6/6	/	2+ = 6/6	/	/	3+ = 6/6
Choice Line 10	Roche	N=5/5	/	1+ = 5/5	/	/	1+ = 1/5<br>2+ = 4/5	2+ = 2/5<br>3+ = 3/5	/	3+ = 5/5
Clinitek Novus	Siemens	N=1/1	/	trace = 1/1	/	1+ = 1/1	/	2+ = 1/1	/	3+ = 1/1
LabStrip U11 Plus	77 Elektronika	N=2/2	/	/	1+ = 2/2	/	2+ = 2/2	/	/	3+ = 2/2
Chronocomb 11 SYS	Analyticon	N=1/1	/	/	1+ = 1/1	/	2+ = 1/1	/	/	3+ = 1/1
Medi-Test Uryxxon 10	Macherey-Nagel	N=1/1	/	/	1+ = 1/1	/	2+ = 1/1	/	/	3+ = 1/1
Combur 10 Test UX	Roche	N=1/1	1+ = 1/1	/	/	/	2+ = 1/1	/	/	3+ = 1/1
Combina 10M	Human	N=2/2	1+ = 2/2	/	/	/	2+ = 2/2	/	/	3+ = 2/2
Combina 13	Human	N=1/1	/	1+ = 1/1	/	2+ = 1/1	/	3+ = 1/1	/	3+ = 1/1
URIN-10	Spinreact	N=1/1	/	/	/	1+ = 1/1	/	2+ = 1/1	/	3+ = 1/1

N - negative. / - this concentration is not measured with this type of urine dipsticks. Shades of grey colour represents degrees of reporting categories from negative to 4+ category.

Table 4. Bilirubin concentrations and assigned categories depending on the urine dipstick type reported by 83 medical laboratories

Urine dipstick type	Manufacturer	Bilirubin concentration (µmol/L)									
		N	8,5	17	34	35	50	51	70	100	103
Combur 10 Test M	Roche	N=25/25	/	1+ = 25/25	/	/	2+ = 25/25	/	/	3+ = 25/25	/
iChem Velocity	Beckman Coulter	N=19/19	N = 7/19<br>trace = 3/19<br>1+ = 9/19	N = 3/19<br>1+ = 14/19<br>2+ = 2/19	1+ = 9/19<br>2+ = 8/19<br>3+ = 2/19	/	1+ = 2/19<br>2+ = 15/19<br>3+ = 1/19<br>4+ = 1/19	/	1+ = 1/19<br>2+ = 12/19<br>3+ = 5/19<br>5+ = 1/19	1+ = 1/19<br>2+ = 1/19<br>3+ = 15/19<br>4+ = 1/19<br>6+ = 1/19	/
Combi Screen 11 SYS plus	Analyticon	N=13/13	/	1+ = 13/13	/	2+ = 13/13	/	/	3+ = 13/13	/	/
Urignost 11	Biognost	N=12/12	/	1+ = 12/12	/	2+ = 12/12	/	/	3+ = 12/12	/	/
Combi Screen 10SL	Analyticon	N=8/8	/	1+ = 8/8	/	2+ = 8/8	/	/	3+ = 8/8	/	/
Clinitek Novus	Siemens	N=1/1	/	1+ = 1/1	/	/	2+ = 1/1	/	3+ = 1/1	/	/
LabStrip U11 Plus	77 Elektronika	N=2/2	/	1+ = 2/2	/	/	/	2+ = 2/2	/	/	3+ = 2/2
Medi-Test Uryxxon 10	Macherey-Nagel	N=1/1	/	1+ = 1/1	/	2+ = 1/1	/	/	3+ = 1/1	/	/
Combina 13	Human	N=1/1	/	1+ = 1/1	/	/	/	2+ = 1/1	/	/	3+ = 1/1
URIN-10	Spinreact	N=1/1	/	1+ = 1/1	/	2+ = 1/1	/	/	3+ = 1/1	/	/

N - negative. / - this concentration is not measured with this type of urine dipsticks. Shades of grey colour represents degrees of reporting categories from negative to 6+ category.

Table 5. Urobilinogen concentrations and assigned categories depending on the urine dipsticks type reported by 110 medical laboratories

Urine dipstick type	Manufacturer	Urobilinogen concentration (µmol/L)														
		N	16	17	33	34	35	50	51	66	70	100	103	131	140	200
Multistix 10 SG	Siemens	N=20/20	N = 14/20<br>trace = 1/20<br>1+ = 5/20	/	1+ = 14/20<br>2+ = 6/20	/	/	/	2+ = 15/20<br>3+ = 5/20	/	/	/	3+ = 19/20<br>4+ = 1/20	/	/	/
Combur 10 Test M	Roche	N=24/24	/	1+ = 24/24	/	/	/	/	1+ = 2/24<br>2+ = 22/24	/	/	/	2+ = 3/24<br>3+ = 21/24	3+ = 12/24<br>4+ = 12/24	/	/
iChem Velocity	Beckman Coulter	N=20/20	/	/	/	N = 5/20<br>1+ = 15/20	/	1+ = 17/20<br>2+ = 3/20	/	/	1+ = 1/20<br>2+ = 16/20<br>3+ = 3/20	2+ = 15/20<br>3+ = 4/20<br>4+ = 1/20	/	/	3+ = 19/20<br>5+ = 1/20	/
Combi Screen 11 SYS plus	Analyticon	N=10/10	/	/	/	/	trace = 2/10<br>1+ = 8/10	/	/	/	1+ = 3/10<br>2+ = 7/10	/	/	/	2+ = 3/10<br>3+ = 7/10	3+ = 9/10<br>4+ = 1/10
Urignost 11	Biognost	N=11/11	/	N=1/11<br>trace = 2/11<br>1+ = 8/11	/	/	1+ = 5/11<br>2+ = 6/11	/	/	/	2+ = 7/11<br>3+ = 4/11	/	/	/	2+ = 1/11<br>3+ = 8/11<br>4+ = 2/11	3+ = 7/11<br>4+ = 4/11
Combi Screen 10SL	Analyticon	N=7/7	/	/	/	/	1+ = 7/7	/	/	/	1+ = 1/7<br>2+ = 6/7	/	/	/	2+ = 2/7<br>3+ = 5/7	3+ = 3/7<br>4+ = 4/7
Combo Stik 10M	DFI co. Ltd	N=3/3	/	/	1+ = 2/3<br>2+ = 1/3	/	/	/	/	2+ = 2/3<br>3+ = 1/3	/	/	/	3+ = 2/3<br>4+ = 1/3	/	/
Choice Line 10	Roche	N=5/5	/	1+ = 5/5	/	/	/	/	/	/	2+ = 5/5	/	/	/	3+ = 5/5	3+ = 3/5<br>4+ = 2/5
Clinitek Novus	Siemens	N=1/1	1+ = 1/1	/	2+ = 1/1	/	/	/	/	3+ = 1/1	/	/	/	4+ = 1/1	/	/
LabStrip U11 Plus	77 Elektronika	N=2/2	/<br>/	/<br>/	1+ = 2/2	/<br>/	/<br>/	/<br>/	/<br>/	/<br>/	2+ = 2/2	/<br>/	/<br>/	/<br>/	3+ = 2/2	3+ = 1/2<br>4+ = 1/2
Chronocomb 11 SYS	Analyticon	N=1/1	/	/	/	/	1+ = 1/1	/	/	/	2+ = 1/1	/	/	/	3+ = 1/1	4+ = 1/1
Medi-Test Uryxxon 10	Macherey-Nagel	N=1/1	/	/	/	/	1+ = 1/1	/	/	/	2+ = 1/1	/	/	/	3+ = 1/1	3+ = 1/1
Combur 10 Test UX	Roche	N=1/1	/	1+ = 1/1	/	/	/	/	/	/	2+ = 1/1	/	/	/	3+ = 1/1	4+ = 1/1
Combina 10M	Human	N=2/2	/<br>/	1+ = 2/2	/<br>/	/<br>/	/<br>/	/<br>/	2+ = 2/2	/<br>/	/<br>/	/<br>/	2+ = 1/2<br>3+ = 1/2	/<br>/	/<br>/	3+ = 1/2<br>4+ = 1/2
Combina 13	Human	N=1/1	/	1+ = 1/1	/	2+ = 1/1	/	/	/	/	2+ = 1/1	/	/	3+ = 1/1	/	/
URIN-10	Spinreact	N=1/1	/	1+ = 1/1	/	/	2+ = 1/1	/	/	/	3+ = 1/1	/	/	/	4+ = 1/1	5+ = 1/1

N - normal. / = this concentration is not measured with this type of urine dipsticks. Shades of grey colour represents degrees of reporting categories from normal to 5+ category.

Table 6. Erythrocyte count and assigned categories depending on the urine dipstick type reported by 84 medical laboratories

Urine dipstick type	Manufacturer	Erythrocyte count (Erc x 10^6/L)												
		N	5-10	10	25	33	50	80	150	163	200	250	300	326
Multistix 10 SG	Siemens	N=21/21	/	N = 1/21<br>trace = 13/21<br>1+ = 7/21	1+ = 20/21<br>2+ = 1/21	/	/	2+ = 20/21<br>3+ = 1/21	/	/	3+ = 21/21	/	/	/
Combur 10 Test M (manual)	Roche	N=17/17	/	1+ =17/17	1+ = 3/17<br>2+ = 14/17	/	2+ = 4/17<br>3+ =13/17	/	/	/	/	3+ = 12/17<br>4+ = 5/17	/	/
Combur 10 Test M (analyzer)	Roche	N=6/6	/	1+ = 6/6	2+ = 6/6	/	3+ = 6/6	/	3+ = 2/6<br>4+ = 4/6	/	/	3+ = 1/6<br>4+ = 1/6<br>5+ = 4/6	/	/
iChem Velocity	Beckman Coulter	N=19/19	/	N = 4/19<br>trace = 10/19<br>1+ = 5/19	/	1+ = 16/19<br>2+ = 3/19	/	/	/	2+ = 16/19<br>3+ = 3/19	/	/	/	3+ = 18/19<br>4+ = 1/19
Combi Screen 11 SYS plus	Analyticon	N=11/11	1+ = 11/11	/	/	/	2+ = 11/11	/	/	/	/	/	3+ = 11/11	/
Combi Screen 10SL	Analyticon	N=7/7	1+ = 7/7	/	/	/	2+ = 7/7	/	/	/	/	/	3+ = 7/7	/
Combo Stik 10M	DFI co. Ltd	N=6/6	/	1+ = 6/6	/	/	2+ = 6/6	/	/	/	/	3+ = 6/6	/	/
Choice Line 10	Roche	N=5/5	1+ = 5/5	1+ = 5/5	1+ = 2/5<br>2+ = 3/5	/	2+ = 3/5<br>3+ = 2/5	/	/	/	/	3+ = 5/5	/	/
Clinitek Novus	Siemens	N=1/1	/	trace = 1/1	1+ = 1/1	/	/	2+ = 1/1	/	/	3+ =1/1	/	/	/
LabStrip U11 Plus	77 Elektronika	N=2/2	1+ = 2/2	/	/	/	2+ = 2/2	/	/	/	/	/	3+ = 2/2	/
Chronocomb 11 SYS	Analyticon	N=1/1	1+ = 1/1	/	/	/	2+ = 1/1	/	/	/	/	/	3+ =1/1	/
Medi-Test Uryxxon 10	Macherey-Nagel	N=1/1	/	1+ = 1/1	/	/	2+ = 1/1	/	/	/	/	3+ =1/1	/	/
Combur 10 Test UX	Roche	N=1/1	1+ = 1/1	/	2+ = 1/1	/	3+ =1/1	/	/	/	/	4+ = 1/1	/	/
Combina 10M	Human	N=2/2	1+ = 2/2	/	/	/	2+ = 2/2	/	/	/	/	3+ = 2/2	/	/
Combina 13	Human	N=1/1	1+ = 1/1	/	/	/	2+ = 1/1	/	/	/	/	3+ =1/1	/	/
Keto-Diastix	Bayer	N=1/1	/	1+ = 1/1	1+ = 1/1	/	/	2+ = 1/1	/	/	3+ =1/1	/	/	/
URIN-10	Spinreact	N=1/1	1+ = 1/1	/	/	/	2+ = 1/1	/	/	/	/	/	/	/

N - negative. / - this concentration is not measured with this type of urine dipsticks. Shades of grey colour represents degrees of reporting categories from negative to 5+ category.

Table 7. Ketones concentration and assigned categories depending on the urine dipstick count reported by 119 medical laboratories

Urine dipstick type	Manufacturer	Ketones concentration (mmol/L)													
		**N**	**0.5**	**1**	**1.5**	**2**	**2.5**	**4**	**5**	**6**	**8**	**10**	**15**	**16**	**30**
Multistix 10 SG	Siemens	N=25/25	N = 2/25<br>trace = 14/25<br>1+ = 9/25	/	1+ = 24/25<br>2+ = 1/25	/	/	2+ = 24/25<br>3+ = 1/25	/	/	2+ = 4/25<br>3+ = 20/25<br>4+ = 1/25	/	/	3+ = 15/25<br>4+ = 10/25	/
Combur 10 Test M (manual)	Roche	N=18/18		1+=18/18	/	/	/	/	2+=18/18	/	/	/	3+=18/18	/	/
Combur 10 Test M (analyzer)	Roche	N=6/6	1+=6/6	/	1+=2/6<br>2+=4/6	/	/	/	2+=2/6<br>3+=4/6	/	/	/	3+=2/6<br>4+=4/6	/	/
iChem Velocity	Beckman Coulter	N=19/19	N = 8/19<br>trace = 8/19<br>1+ = 3/19	N = 1/19<br>1+ = 18/19	/	1+ = 16/19<br>2+ = 3/19	/	1+ = 2/19<br>2+ = 15/19<br>3+ = 2/19	/	1+ = 1/19<br>2+ = 16/19<br>4+ = 2/19	2+ = 3/19<br>3+ = 14/19<br>4+ = 2/19	3+ = 17/19<br>4+ = 2/19	3+ = 12/19<br>4+ = 7/19	3+ = 12/19<br>4+ = 7/19	/
Combi Screen 11 SYS plus	Analyticon	N=13/13	/	trace = 8/13<br>1+ = 5/13	/	/	1+ = 13/13	/	/	/	/	2+ = 13/13	/	/	3+ = 13/13
Urignost 11	Biognost	N=11/11	trace = 7/11<br>1+ = 4/11	/	1+ = 10/11<br>2+ = 1/11	/	/	2+ = 11/11	/	/	2+ = 1/11<br>3+ = 10/11	/	/	3+ = 8/11<br>4+ = 3/11	/
Combi Screen 10SL	Analyticon	N=8/8	/	trace = 3/8<br>1+ = 5/8	/	/	1+ = 7/8<br>2+ = 1/8	/	/	/	/	2+ = 7/8<br>3+ = 1/8	/	/	3+ = 8/8
Combo Stik 10M	DFI co. Ltd	N=5/5	/	/	1+ = 5/5	/	/	2+ = 5/5	/	/	/	3+ = 5/5	/	/	/
Choice Line 10	Roche	N=4/4	1+ = 4/4	/	2+ = 4/4	/	/	/	3+ = 4/4	/	/	/	3+ = 4/4	/	/
Clinitek Novus	Siemens	N=1/1	trace = 1/1	/	1+ = 1/1	/	/	2+ = 1/1	/	/	3+ = 1/1	/	4+ = 1/1	/	/
LabStrip U11 Plus	77 Elektronika	N=2/2	trace = 2/2	/	1+ = 2/2	/	/	/	2+ = 2/2	/	/	/	3+ = 2/2	/	/
Medi-Test Uryxxon 10	Macherey-Nagel	N=1/1	/	/	/	/	1+ = 1/1	/	/	/	/	2+ = 1/1	/	/	3+ = 1/1
Combur 10 Test UX	Roche	N=1/1	/	1+ = 1/1	/	/	/	/	2+ = 1/1	/	/	/	3+ = 1/1	/	/
Combina 10M	Human	N=2/2	/	/	1+ = 2/2	/	/	/	2+ = 2/2	/	/	/	3+ = 2/2	/	/
Combina 13	Human	N=1/1	1+ = 1/1	/	1+ = 1/1	/	/	2+ = 1/1	/	/	3+ = 1/1	/	/	/	/
Keto-Diastix	Bayer	N=1/1	trace = 1/1	/	1+ = 1/1	/	/	2+ = 1/1	/	/	3+ = 1/1	/	/	3+ = 1/1	
URIN-10	Spinreact	N=1/1	1+ = 1/1	/	1+ = 1/1	/	/	2+ = 1/1	/	/	3+ = 1/1	/	/	4+ = 1/1	/

N - negative. / - this concentration is not measured with this type of urine dipsticks. Shades of grey colour represents degrees of reporting categories from negative to 4+ category.
